# μ-Oxalato-κ^4^ *O* ^1^,*O* ^2^:*O* ^1′^,*O* ^2′^-bis­[diaqua­(2,2′-bipyridyl-κ^2^ *N*,*N*′)zinc] bis­[2-(1*H*-benzotriazol-1-yl)acetate] hexa­hydrate

**DOI:** 10.1107/S1600536812001778

**Published:** 2012-01-21

**Authors:** Ling Zeng, Qinglin Wang

**Affiliations:** a College of Chemistry and Chemical Engineering of Bohai University, Jinzhou, Liaoning 121000, People’s Republic of China

## Abstract

The asymmetric unit of the title compound, [Zn_2_(C_2_O_4_)(C_10_H_8_N_2_)_2_(H_2_O)_4_](C_8_H_6_N_3_O_2_)_2_·6H_2_O, contains one half of the centrosymmetric binuclear cation, one anion and three water mol­ecules. In the cation, the oxalate ligand bridges two Zn^II^ ions in a bis-bidentate fashion, so each Zn^II^ ion is coordinated by two O atoms from the oxalate ligand, two N atoms from two 2,2′-bipyridine ligands and two water mol­ecules in a distorted octa­hedral arrangement. The mean planes of the oxalate and 2,2′-bipyridine ligands form a dihedral angle of 80.0 (1)°. An extensive three-dimensional hydrogen-bonding network formed by classical O—H⋯O and O—H⋯N inter­actions consolidates the crystal packing.

## Related literature

For applications of oxalate complexes, see: Decurtins *et al.* (1994 ▶); Liu *et al.* (2009 ▶). For related structures, see: Sun *et al.* (2009 ▶); Zheng *et al.* (2010 ▶).
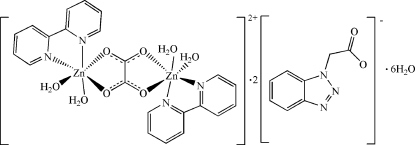


## Experimental

### 

#### Crystal data

[Zn_2_(C_2_O_4_)(C_10_H_8_N_2_)_2_(H_2_O)_4_](C_8_H_6_N_3_O_2_)_2_·6H_2_O
*M* *_r_* = 1063.60
Monoclinic, 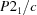

*a* = 16.791 (2) Å
*b* = 18.218 (2) Å
*c* = 7.7461 (9) Å
β = 92.233 (2)°
*V* = 2367.7 (5) Å^3^
*Z* = 2
Mo *K*α radiation
μ = 1.10 mm^−1^
*T* = 295 K
0.22 × 0.19 × 0.17 mm

#### Data collection

Bruker APEXII CCD area-detector diffractometer
Absorption correction: multi-scan (*SADABS*; Sheldrick, 1996 ▶) *T*_min_ = 0.795, *T*_max_ = 0.836
12364 measured reflections
4193 independent reflections
2281 reflections with *I* > 2σ(*I*)
*R*_int_ = 0.092

#### Refinement

*R*[*F* ^2^ > 2σ(*F* ^2^)] = 0.054
*wR*(*F* ^2^) = 0.111
*S* = 1.00
4193 reflections
307 parameters
H-atom parameters constrained
Δρ_max_ = 0.36 e Å^−3^
Δρ_min_ = −0.34 e Å^−3^

### 

Data collection: *APEX2* (Bruker, 2005 ▶); cell refinement: *SAINT* (Bruker, 2005 ▶); data reduction: *SAINT*; program(s) used to solve structure: *SHELXS97* (Sheldrick, 2008 ▶); program(s) used to refine structure: *SHELXL97* (Sheldrick, 2008 ▶); molecular graphics: *SHELXTL* (Sheldrick, 2008 ▶); software used to prepare material for publication: *SHELXTL*.

## Supplementary Material

Crystal structure: contains datablock(s) global, I. DOI: 10.1107/S1600536812001778/cv5232sup1.cif

Structure factors: contains datablock(s) I. DOI: 10.1107/S1600536812001778/cv5232Isup2.hkl

Additional supplementary materials:  crystallographic information; 3D view; checkCIF report

## Figures and Tables

**Table 1:** Hydrogen-bond geometry (Å, °)

*D*—H⋯*A*	*D*—H	H⋯*A*	*D*⋯*A*	*D*—H⋯*A*
O7—H24⋯N5^i^	0.85	2.11	2.924 (6)	160
O6—H22⋯O7^ii^	0.85	2.11	2.859 (5)	146
O1—H2⋯O2^iii^	0.85	1.96	2.755 (4)	155
O7—H23⋯O9^iv^	0.85	1.92	2.748 (5)	166
O3—H3⋯O9^iv^	0.85	1.87	2.718 (4)	177
O1—H1⋯O8^iv^	0.85	1.85	2.692 (4)	171
O6—H21⋯O7	0.85	2.05	2.860 (5)	160
O5—H19⋯O6	0.85	1.88	2.728 (5)	178
O5—H20⋯O8	0.85	2.08	2.928 (5)	174
O3—H4⋯O5	0.85	1.83	2.678 (4)	172

Symmetry codes: (i) 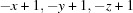; (ii) 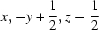; (iii) 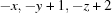; (iv) 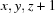.

**Fig. 1.**
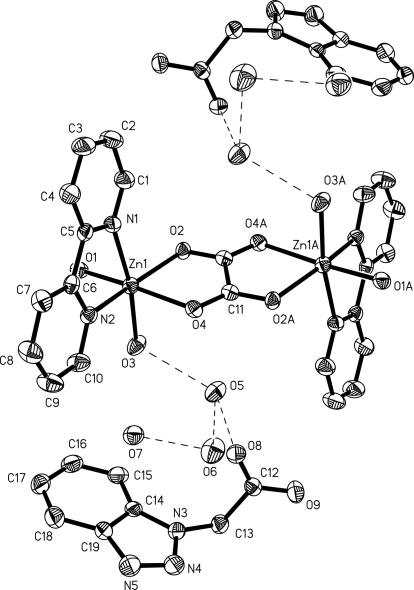
A portion of the crystal structure of (I) showing the atomic labeling and 30% probability displacement ellopsoids. Unlabelled atoms, and those with letter A in labels, are related to the labelled ones by symmetry [ (A) -x, -y + 1, -z + 2]. Dashed lines denote hydrogen bonds, H atoms omitted for clarity.

## supplementary crystallographic information

### Comment

The metal oxalate compounds are studied as molecular-based magnets, thermally stable dielectrics and open framework structures (Decurtins *et al.*, 1994; Liu *et al.*, 2009). The flexible ligand 2-(1*H*-benzo[*d*][1,2,3]triazol-1-yl)acetic acid containing a carboxylate group can be used to construct MOFs (Zheng *et al.*, 2010). We report here the synthesis and crystal structure of the title complex (I).

The asymmetric unit of (I), [Zn_2_(C_2_O_4_)(C_10_H_8_N_2_)_2_(H_2_O)_4_]^2+^. 2(C_8_H_6_N_3_O_2_)^-^.6(H_2_O), contains one half of the centrosymmetric binuclear cation, one anion and three lattice water molecules (Fig.1). In the cation, the oxalato ligand bridges two Zn^II^ ions in a bis-bidentate fashion, so each Zn center is coordinated by two O atoms from the oxalato ligand, two N atoms from two 2,2'-bipyridine ligands and two water molecules in a distorted octahedral arrangement, in which the basal plane is formed by O1, O2, O4 and N2 with a mean deviation of 0.1880 (1) Å from the least-squares plane. The axial positions are occupied by N1 and O3 with an N1—Zn1—O3 angle of 170.72 (14) °. The Zn—O and Zn—N bond distances fall in the range 2.050 (3) - 2.171 (3) Å. The deprotonated bis(bidentate) oxalate group is coordinated to two zinc with the distance between Zn1 and Zn1A being 5.568 (3) Å, which compares well with similar binuclear oxalate-bridged complexes (Sun *et al.*, 2009). The mean planes of the oxalato and 2,2'-bipyridine ligands form a dihedral angle of 80.0 (1)°. An extensive three-dimensional hydrogen-bonding network formed by classical O—H···O and O—H···N interactions (Table 1) consolidate the crystal packing.

### Experimental

A mixture of Zn(Ac)_2_ (0.5 mmol), 2-(1*H*-benzo[*d*][1,2,3]triazol-1-yl)acetic acid (0.5 mmol), 2,2'-bipyridine(0.5 mmol) and oxalic acid (0.25 mmol) was dissolved in water (30 ml) and methanol (10 ml). and the pH of the solution was adjusted to 6–7 with 0.2 *M* aqueous NaOH and the solution was stirred for 3 h at room temperature. The solution was flitered and the flitrate was allowed to stand at room temperature. After slow evaporation over 3 weeks, colourless block single crystals were obtained.

### Refinement

All H atoms were placed in idealized positions (O—H = 0.85 Å and C—H = 0.93–0.97 Å) and refined as riding atoms, with *U*_iso_(H) = 1.2*U*_eq_(C) and *U*_iso_(H) = 1.5*U*_eq_(O).

### Crystal data

**Table:**

[Zn_2_(C_2_O_4_)(C_10_H_8_N_2_)_2_(H_2_O)_4_](C_8_H_6_N_3_O_2_)_2_·6H_2_O	*F*(000) = 1100
*M**_r_* = 1063.60	*D*_x_ = 1.492 Mg m^−^^3^
Monoclinic, *P*2_1_/*c*	Mo *K*α radiation, λ = 0.71073 Å
Hall symbol: -P 2ybc	Cell parameters from 721 reflections
*a* = 16.791 (2) Å	θ = 2.4–16.9°
*b* = 18.218 (2) Å	µ = 1.10 mm^−^^1^
*c* = 7.7461 (9) Å	*T* = 295 K
β = 92.233 (2)°	Block, colourless
*V* = 2367.7 (5) Å^3^	0.22 × 0.19 × 0.17 mm
*Z* = 2	

### Data collection

**Table:**

Bruker APEXII CCD area-detector diffractometer	4193 independent reflections
Radiation source: fine-focus sealed tube	2281 reflections with *I* > 2σ(*I*)
graphite	*R*_int_ = 0.092
φ and ω scans	θ_max_ = 25.1°, θ_min_ = 1.2°
Absorption correction: multi-scan (*SADABS*; Sheldrick, 1996)	*h* = −19→19
*T*_min_ = 0.795, *T*_max_ = 0.836	*k* = −14→21
12364 measured reflections	*l* = −8→9

### Refinement

**Table:**

Refinement on *F*^2^	Primary atom site location: structure-invariant direct methods
Least-squares matrix: full	Secondary atom site location: difference Fourier map
*R*[*F*^2^ > 2σ(*F*^2^)] = 0.054	Hydrogen site location: inferred from neighbouring sites
*wR*(*F*^2^) = 0.111	H-atom parameters constrained
*S* = 1.00	*w* = 1/[σ^2^(*F*_o_^2^) + (0.0272*P*)^2^ + 0.0437*P*] where *P* = (*F*_o_^2^ + 2*F*_c_^2^)/3
4193 reflections	(Δ/σ)_max_ = 0.001
307 parameters	Δρ_max_ = 0.36 e Å^−^^3^
0 restraints	Δρ_min_ = −0.34 e Å^−^^3^

### Special details

**Table:**

Geometry. All e.s.d.'s (except the e.s.d. in the dihedral angle between two l.s. planes) are estimated using the full covariance matrix. The cell e.s.d.'s are taken into account individually in the estimation of e.s.d.'s in distances, angles and torsion angles; correlations between e.s.d.'s in cell parameters are only used when they are defined by crystal symmetry. An approximate (isotropic) treatment of cell e.s.d.'s is used for estimating e.s.d.'s involving l.s. planes.
Refinement. Refinement of *F*^2^ against ALL reflections. The weighted *R*-factor *wR* and goodness of fit *S* are based on *F*^2^, conventional *R*-factors *R* are based on *F*, with *F* set to zero for negative *F*^2^. The threshold expression of *F*^2^ > σ(*F*^2^) is used only for calculating *R*-factors(gt) *etc*. and is not relevant to the choice of reflections for refinement. *R*-factors based on *F*^2^ are statistically about twice as large as those based on *F*, and *R*- factors based on ALL data will be even larger.

### Fractional atomic coordinates and isotropic or equivalent isotropic displacement parameters (Å^2^)

**Table:**

	*x*	*y*	*z*	*U*_iso_*/*U*_eq_	
Zn1	0.08736 (3)	0.56912 (3)	0.76609 (7)	0.03784 (19)	
O1	0.10766 (17)	0.54817 (17)	1.0267 (4)	0.0483 (9)	
H1	0.1536	0.5424	1.0748	0.072*	
H2	0.0745	0.5505	1.1068	0.072*	
O2	−0.00201 (17)	0.48940 (17)	0.7246 (4)	0.0383 (8)	
O3	0.18133 (18)	0.49957 (18)	0.7310 (4)	0.0555 (10)	
H3	0.2113	0.4760	0.8025	0.083*	
H4	0.1885	0.4809	0.6322	0.083*	
O4	0.07157 (17)	0.56750 (17)	0.4867 (4)	0.0390 (8)	
O5	0.1949 (2)	0.4297 (2)	0.4293 (4)	0.0653 (11)	
H20	0.2112	0.4586	0.3520	0.098*	
H19	0.2295	0.3957	0.4355	0.098*	
O6	0.3061 (2)	0.3209 (2)	0.4586 (5)	0.0900 (14)	
H21	0.3154	0.3293	0.5654	0.135*	
H22	0.3118	0.2745	0.4541	0.135*	
O7	0.3566 (2)	0.31526 (19)	0.8153 (4)	0.0694 (11)	
H23	0.3347	0.3469	0.8786	0.104*	
H24	0.4063	0.3241	0.8240	0.104*	
O8	0.25638 (19)	0.5197 (2)	0.1510 (4)	0.0537 (10)	
O9	0.27760 (19)	0.4289 (2)	−0.0328 (4)	0.0565 (10)	
N1	0.0028 (2)	0.6530 (2)	0.8248 (5)	0.0403 (10)	
N2	0.1533 (2)	0.6665 (2)	0.7435 (5)	0.0389 (10)	
N3	0.4175 (2)	0.5278 (2)	0.2341 (5)	0.0464 (11)	
N4	0.4561 (3)	0.5771 (3)	0.1360 (5)	0.0610 (13)	
N5	0.4813 (3)	0.6316 (3)	0.2316 (6)	0.0641 (14)	
C1	−0.0722 (3)	0.6424 (3)	0.8689 (7)	0.0564 (15)	
H1A	−0.0907	0.5944	0.8752	0.068*	
C2	−0.1237 (3)	0.6982 (3)	0.9056 (8)	0.0672 (18)	
H2A	−0.1756	0.6887	0.9371	0.081*	
C3	−0.0953 (3)	0.7691 (3)	0.8937 (7)	0.0691 (18)	
H3A	−0.1284	0.8088	0.9154	0.083*	
C4	−0.0182 (3)	0.7806 (3)	0.8497 (7)	0.0583 (16)	
H4A	0.0016	0.8281	0.8423	0.070*	
C5	0.0301 (3)	0.7216 (3)	0.8165 (6)	0.0396 (13)	
C6	0.1144 (3)	0.7296 (3)	0.7702 (5)	0.0371 (12)	
C7	0.1519 (3)	0.7963 (3)	0.7562 (6)	0.0530 (15)	
H7	0.1242	0.8392	0.7782	0.064*	
C8	0.2308 (3)	0.8002 (3)	0.7098 (7)	0.0622 (16)	
H8	0.2564	0.8452	0.6995	0.075*	
C9	0.2693 (3)	0.7360 (3)	0.6799 (7)	0.0671 (17)	
H9	0.3221	0.7363	0.6479	0.080*	
C10	0.2292 (3)	0.6708 (3)	0.6975 (7)	0.0551 (15)	
H10	0.2563	0.6274	0.6763	0.066*	
C11	0.0221 (3)	0.5225 (3)	0.4331 (6)	0.0340 (12)	
C12	0.2985 (3)	0.4722 (3)	0.0863 (6)	0.0417 (13)	
C13	0.3843 (3)	0.4627 (3)	0.1566 (7)	0.0503 (14)	
H13A	0.3857	0.4237	0.2419	0.060*	
H13B	0.4173	0.4477	0.0628	0.060*	
C14	0.4181 (3)	0.5520 (3)	0.4017 (6)	0.0436 (14)	
C15	0.3893 (3)	0.5221 (3)	0.5522 (7)	0.0544 (15)	
H15	0.3615	0.4780	0.5526	0.065*	
C16	0.4046 (3)	0.5618 (4)	0.6991 (7)	0.0648 (17)	
H16	0.3867	0.5441	0.8033	0.078*	
C17	0.4460 (3)	0.6276 (4)	0.6982 (8)	0.0690 (19)	
H17	0.4556	0.6522	0.8022	0.083*	
C18	0.4729 (3)	0.6575 (3)	0.5508 (8)	0.0623 (17)	
H18	0.4992	0.7024	0.5512	0.075*	
C19	0.4590 (3)	0.6175 (3)	0.3982 (7)	0.0494 (14)	

### Atomic displacement parameters (Å^2^)

**Table:**

	*U*^11^	*U*^22^	*U*^33^	*U*^12^	*U*^13^	*U*^23^
Zn1	0.0404 (3)	0.0329 (3)	0.0401 (3)	0.0014 (3)	0.0005 (2)	−0.0017 (3)
O1	0.0378 (19)	0.069 (3)	0.0375 (19)	0.0022 (18)	−0.0002 (16)	0.0059 (18)
O2	0.0428 (19)	0.042 (2)	0.0297 (19)	−0.0046 (17)	−0.0003 (15)	0.0015 (16)
O3	0.067 (2)	0.058 (3)	0.041 (2)	0.027 (2)	−0.0024 (18)	−0.0063 (18)
O4	0.0385 (18)	0.0355 (19)	0.043 (2)	−0.0073 (18)	0.0026 (15)	0.0050 (17)
O5	0.077 (3)	0.071 (3)	0.049 (2)	0.011 (2)	0.0104 (19)	−0.001 (2)
O6	0.108 (3)	0.070 (3)	0.091 (3)	0.029 (3)	−0.004 (3)	−0.006 (2)
O7	0.070 (3)	0.059 (3)	0.080 (3)	−0.004 (2)	0.014 (2)	−0.008 (2)
O8	0.045 (2)	0.059 (3)	0.057 (2)	0.012 (2)	−0.0001 (18)	−0.011 (2)
O9	0.056 (2)	0.054 (2)	0.058 (2)	0.010 (2)	−0.0149 (18)	−0.014 (2)
N1	0.037 (2)	0.040 (3)	0.044 (3)	0.003 (2)	0.001 (2)	−0.002 (2)
N2	0.044 (3)	0.034 (3)	0.039 (2)	−0.002 (2)	0.005 (2)	−0.002 (2)
N3	0.041 (2)	0.060 (3)	0.038 (3)	0.001 (3)	0.000 (2)	−0.002 (3)
N4	0.059 (3)	0.084 (4)	0.041 (3)	−0.016 (3)	0.005 (2)	0.007 (3)
N5	0.066 (3)	0.076 (4)	0.051 (3)	−0.012 (3)	0.007 (3)	0.003 (3)
C1	0.046 (3)	0.051 (4)	0.073 (4)	0.001 (3)	0.003 (3)	−0.011 (3)
C2	0.041 (3)	0.061 (4)	0.101 (5)	−0.004 (3)	0.013 (3)	−0.016 (4)
C3	0.051 (4)	0.061 (4)	0.096 (5)	0.015 (3)	0.010 (3)	−0.021 (4)
C4	0.050 (3)	0.038 (3)	0.086 (4)	0.002 (3)	0.002 (3)	−0.018 (3)
C5	0.043 (3)	0.035 (3)	0.040 (3)	0.002 (3)	0.000 (2)	−0.009 (3)
C6	0.047 (3)	0.033 (3)	0.031 (3)	0.001 (3)	−0.003 (2)	0.001 (2)
C7	0.059 (4)	0.040 (3)	0.059 (4)	−0.004 (3)	0.001 (3)	0.004 (3)
C8	0.057 (4)	0.043 (4)	0.087 (5)	−0.017 (3)	0.009 (3)	0.011 (3)
C9	0.048 (4)	0.059 (4)	0.095 (5)	−0.004 (3)	0.017 (3)	0.010 (4)
C10	0.051 (4)	0.043 (4)	0.072 (4)	0.005 (3)	0.015 (3)	0.001 (3)
C11	0.035 (3)	0.025 (3)	0.042 (3)	0.004 (2)	0.001 (2)	0.007 (2)
C12	0.041 (3)	0.047 (4)	0.036 (3)	−0.001 (3)	−0.004 (3)	0.009 (3)
C13	0.048 (3)	0.055 (4)	0.048 (3)	0.008 (3)	−0.002 (3)	−0.009 (3)
C14	0.030 (3)	0.066 (4)	0.034 (3)	0.005 (3)	−0.001 (2)	0.001 (3)
C15	0.043 (3)	0.071 (4)	0.049 (4)	0.004 (3)	0.007 (3)	0.007 (3)
C16	0.052 (4)	0.095 (5)	0.048 (4)	0.007 (4)	0.011 (3)	0.000 (4)
C17	0.049 (4)	0.108 (6)	0.049 (4)	0.020 (4)	−0.010 (3)	−0.020 (4)
C18	0.053 (4)	0.069 (4)	0.065 (4)	0.001 (3)	−0.007 (3)	−0.009 (4)
C19	0.039 (3)	0.062 (4)	0.047 (4)	0.002 (3)	−0.001 (3)	−0.009 (3)

### Geometric parameters (Å, °)

**Table:**

Zn1—O3	2.050 (3)	C2—C3	1.381 (7)
Zn1—O1	2.070 (3)	C2—H2A	0.9300
Zn1—N2	2.102 (4)	C3—C4	1.368 (7)
Zn1—O2	2.104 (3)	C3—H3A	0.9300
Zn1—N1	2.147 (4)	C4—C5	1.376 (6)
Zn1—O4	2.171 (3)	C4—H4A	0.9300
O1—H1	0.8501	C5—C6	1.481 (6)
O1—H2	0.8500	C6—C7	1.374 (6)
O2—C11^i^	1.273 (5)	C7—C8	1.387 (7)
O3—H3	0.8500	C7—H7	0.9300
O3—H4	0.8500	C8—C9	1.361 (7)
O4—C11	1.229 (5)	C8—H8	0.9300
O5—H20	0.8500	C9—C10	1.374 (7)
O5—H19	0.8501	C9—H9	0.9300
O6—H21	0.8501	C10—H10	0.9300
O6—H22	0.8499	C11—O2^i^	1.273 (5)
O7—H23	0.8499	C11—C11^i^	1.534 (9)
O7—H24	0.8500	C12—C13	1.530 (6)
O8—C12	1.236 (6)	C13—H13A	0.9700
O9—C12	1.253 (5)	C13—H13B	0.9700
N1—C1	1.332 (6)	C14—C19	1.377 (7)
N1—C5	1.333 (6)	C14—C15	1.390 (6)
N2—C10	1.339 (6)	C15—C16	1.364 (7)
N2—C6	1.343 (6)	C15—H15	0.9300
N3—N4	1.357 (5)	C16—C17	1.386 (8)
N3—C14	1.371 (6)	C16—H16	0.9300
N3—C13	1.433 (6)	C17—C18	1.358 (7)
N4—N5	1.300 (6)	C17—H17	0.9300
N5—C19	1.381 (6)	C18—C19	1.401 (7)
C1—C2	1.371 (7)	C18—H18	0.9300
C1—H1A	0.9300		
			
O3—Zn1—O1	85.24 (12)	N1—C5—C4	121.1 (5)
O3—Zn1—N2	95.73 (15)	N1—C5—C6	116.0 (4)
O1—Zn1—N2	99.77 (13)	C4—C5—C6	122.9 (5)
O3—Zn1—O2	95.81 (13)	N2—C6—C7	121.1 (5)
O1—Zn1—O2	96.37 (12)	N2—C6—C5	115.4 (4)
N2—Zn1—O2	160.85 (13)	C7—C6—C5	123.5 (5)
O3—Zn1—N1	170.72 (14)	C6—C7—C8	120.8 (5)
O1—Zn1—N1	90.57 (13)	C6—C7—H7	119.6
N2—Zn1—N1	76.80 (16)	C8—C7—H7	119.6
O2—Zn1—N1	92.88 (14)	C9—C8—C7	117.6 (5)
O3—Zn1—O4	85.65 (11)	C9—C8—H8	121.2
O1—Zn1—O4	168.31 (12)	C7—C8—H8	121.2
N2—Zn1—O4	88.43 (13)	C8—C9—C10	119.3 (5)
O2—Zn1—O4	77.28 (11)	C8—C9—H9	120.4
N1—Zn1—O4	99.48 (13)	C10—C9—H9	120.4
Zn1—O1—H1	124.2	N2—C10—C9	123.4 (5)
Zn1—O1—H2	128.0	N2—C10—H10	118.3
H1—O1—H2	107.2	C9—C10—H10	118.3
C11^i^—O2—Zn1	115.1 (3)	O4—C11—O2^i^	126.0 (4)
Zn1—O3—H3	131.7	O4—C11—C11^i^	117.8 (6)
Zn1—O3—H4	120.2	O2^i^—C11—C11^i^	116.2 (6)
H3—O3—H4	106.5	O8—C12—O9	126.2 (5)
C11—O4—Zn1	113.6 (3)	O8—C12—C13	118.7 (5)
H20—O5—H19	104.5	O9—C12—C13	115.1 (5)
H21—O6—H22	101.7	N3—C13—C12	113.6 (4)
H23—O7—H24	105.8	N3—C13—H13A	108.8
C1—N1—C5	118.6 (5)	C12—C13—H13A	108.8
C1—N1—Zn1	126.2 (4)	N3—C13—H13B	108.8
C5—N1—Zn1	115.2 (3)	C12—C13—H13B	108.8
C10—N2—C6	117.7 (4)	H13A—C13—H13B	107.7
C10—N2—Zn1	125.6 (4)	N3—C14—C19	104.2 (4)
C6—N2—Zn1	116.7 (3)	N3—C14—C15	132.7 (5)
N4—N3—C14	109.4 (4)	C19—C14—C15	123.1 (5)
N4—N3—C13	120.0 (4)	C16—C15—C14	115.6 (6)
C14—N3—C13	130.7 (5)	C16—C15—H15	122.2
N5—N4—N3	109.8 (4)	C14—C15—H15	122.2
N4—N5—C19	107.2 (5)	C15—C16—C17	122.2 (6)
N1—C1—C2	123.7 (5)	C15—C16—H16	118.9
N1—C1—H1A	118.1	C17—C16—H16	118.9
C2—C1—H1A	118.1	C18—C17—C16	122.3 (6)
C1—C2—C3	117.2 (5)	C18—C17—H17	118.9
C1—C2—H2A	121.4	C16—C17—H17	118.9
C3—C2—H2A	121.4	C17—C18—C19	116.8 (6)
C4—C3—C2	119.4 (5)	C17—C18—H18	121.6
C4—C3—H3A	120.3	C19—C18—H18	121.6
C2—C3—H3A	120.3	C14—C19—N5	109.5 (5)
C3—C4—C5	119.9 (5)	C14—C19—C18	120.0 (5)
C3—C4—H4A	120.1	N5—C19—C18	130.5 (6)
C5—C4—H4A	120.1		
			
O3—Zn1—O2—C11^i^	−83.7 (3)	C3—C4—C5—N1	0.7 (8)
O1—Zn1—O2—C11^i^	−169.5 (3)	C3—C4—C5—C6	−179.3 (5)
N2—Zn1—O2—C11^i^	43.1 (6)	C10—N2—C6—C7	−2.1 (7)
N1—Zn1—O2—C11^i^	99.6 (3)	Zn1—N2—C6—C7	−179.2 (3)
O4—Zn1—O2—C11^i^	0.5 (3)	C10—N2—C6—C5	178.6 (4)
O3—Zn1—O4—C11	95.5 (3)	Zn1—N2—C6—C5	1.5 (5)
O1—Zn1—O4—C11	56.6 (7)	N1—C5—C6—N2	−0.6 (6)
N2—Zn1—O4—C11	−168.7 (3)	C4—C5—C6—N2	179.4 (4)
O2—Zn1—O4—C11	−1.5 (3)	N1—C5—C6—C7	−179.9 (4)
N1—Zn1—O4—C11	−92.3 (3)	C4—C5—C6—C7	0.1 (8)
O1—Zn1—N1—C1	−78.5 (4)	N2—C6—C7—C8	1.6 (7)
N2—Zn1—N1—C1	−178.4 (4)	C5—C6—C7—C8	−179.0 (5)
O2—Zn1—N1—C1	18.0 (4)	C6—C7—C8—C9	−0.4 (8)
O4—Zn1—N1—C1	95.5 (4)	C7—C8—C9—C10	−0.3 (9)
O1—Zn1—N1—C5	100.9 (3)	C6—N2—C10—C9	1.4 (8)
N2—Zn1—N1—C5	1.0 (3)	Zn1—N2—C10—C9	178.2 (4)
O2—Zn1—N1—C5	−162.7 (3)	C8—C9—C10—N2	−0.2 (9)
O4—Zn1—N1—C5	−85.1 (3)	Zn1—O4—C11—O2^i^	−179.8 (4)
O3—Zn1—N2—C10	7.4 (4)	Zn1—O4—C11—C11^i^	2.1 (6)
O1—Zn1—N2—C10	93.5 (4)	N4—N3—C13—C12	−89.2 (5)
O2—Zn1—N2—C10	−119.4 (5)	C14—N3—C13—C12	91.0 (6)
N1—Zn1—N2—C10	−178.2 (4)	O8—C12—C13—N3	−25.8 (7)
O4—Zn1—N2—C10	−78.1 (4)	O9—C12—C13—N3	155.1 (4)
O3—Zn1—N2—C6	−175.8 (3)	N4—N3—C14—C19	−0.2 (5)
O1—Zn1—N2—C6	−89.6 (3)	C13—N3—C14—C19	179.6 (5)
O2—Zn1—N2—C6	57.4 (6)	N4—N3—C14—C15	−178.1 (5)
N1—Zn1—N2—C6	−1.3 (3)	C13—N3—C14—C15	1.6 (9)
O4—Zn1—N2—C6	98.8 (3)	N3—C14—C15—C16	177.1 (5)
C14—N3—N4—N5	−0.1 (6)	C19—C14—C15—C16	−0.5 (7)
C13—N3—N4—N5	−179.9 (4)	C14—C15—C16—C17	0.2 (8)
N3—N4—N5—C19	0.4 (6)	C15—C16—C17—C18	1.0 (9)
C5—N1—C1—C2	0.5 (8)	C16—C17—C18—C19	−1.8 (8)
Zn1—N1—C1—C2	179.8 (4)	N3—C14—C19—N5	0.4 (5)
N1—C1—C2—C3	0.7 (9)	C15—C14—C19—N5	178.6 (5)
C1—C2—C3—C4	−1.1 (9)	N3—C14—C19—C18	−178.5 (4)
C2—C3—C4—C5	0.5 (9)	C15—C14—C19—C18	−0.3 (8)
C1—N1—C5—C4	−1.1 (7)	N4—N5—C19—C14	−0.5 (6)
Zn1—N1—C5—C4	179.4 (4)	N4—N5—C19—C18	178.3 (5)
C1—N1—C5—C6	178.8 (4)	C17—C18—C19—C14	1.4 (8)
Zn1—N1—C5—C6	−0.6 (5)	C17—C18—C19—N5	−177.2 (5)

Symmetry codes: (i) −*x*, −*y*+1, −*z*+1.

### Hydrogen-bond geometry (Å, °)

**Table:**

*D*—H···*A*	*D*—H	H···*A*	*D*···*A*	*D*—H···*A*
O7—H24···N5^ii^	0.85	2.11	2.924 (6)	160
O6—H22···O7^iii^	0.85	2.11	2.859 (5)	146
O1—H2···O2^iv^	0.85	1.96	2.755 (4)	155
O7—H23···O9^v^	0.85	1.92	2.748 (5)	166
O3—H3···O9^v^	0.85	1.87	2.718 (4)	177
O1—H1···O8^v^	0.85	1.85	2.692 (4)	171
O6—H21···O7	0.85	2.05	2.860 (5)	160
O5—H19···O6	0.85	1.88	2.728 (5)	178
O5—H20···O8	0.85	2.08	2.928 (5)	174
O3—H4···O5	0.85	1.83	2.678 (4)	172

Symmetry codes: (ii) −*x*+1, −*y*+1, −*z*+1; (iii) *x*, −*y*+1/2, *z*−1/2; (iv) −*x*, −*y*+1, −*z*+2; (v) *x*, *y*, *z*+1.
